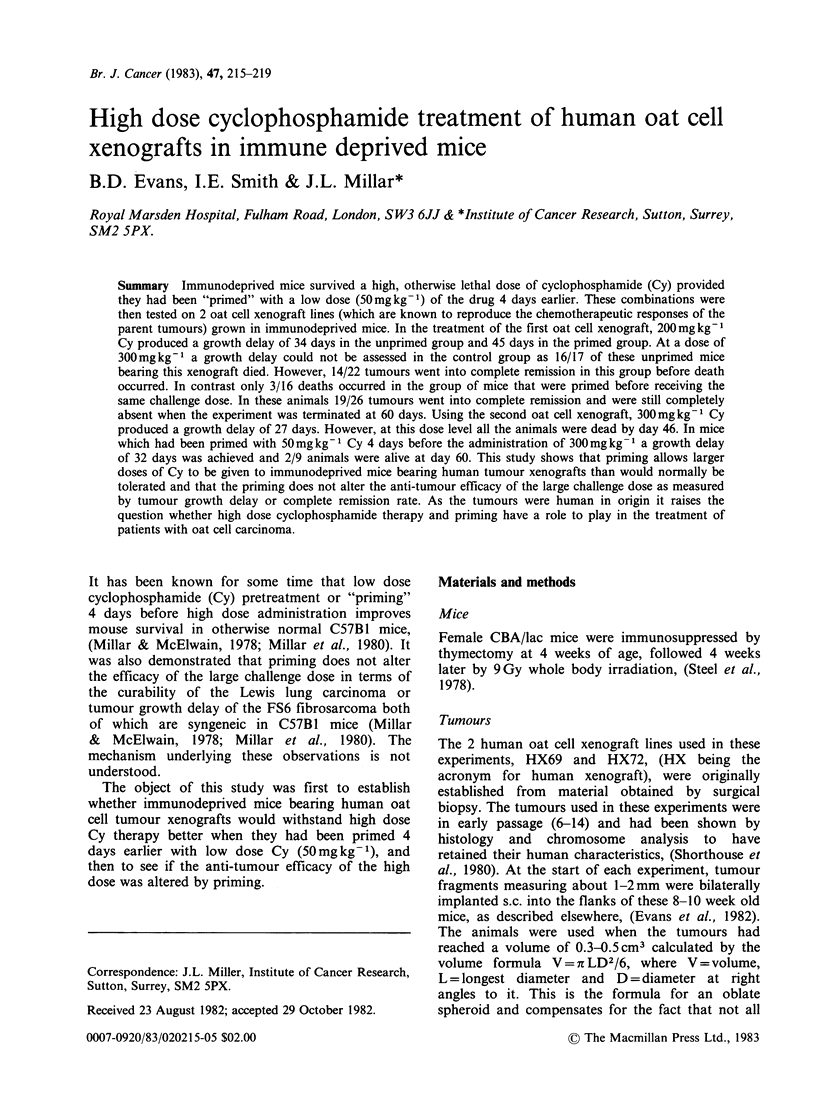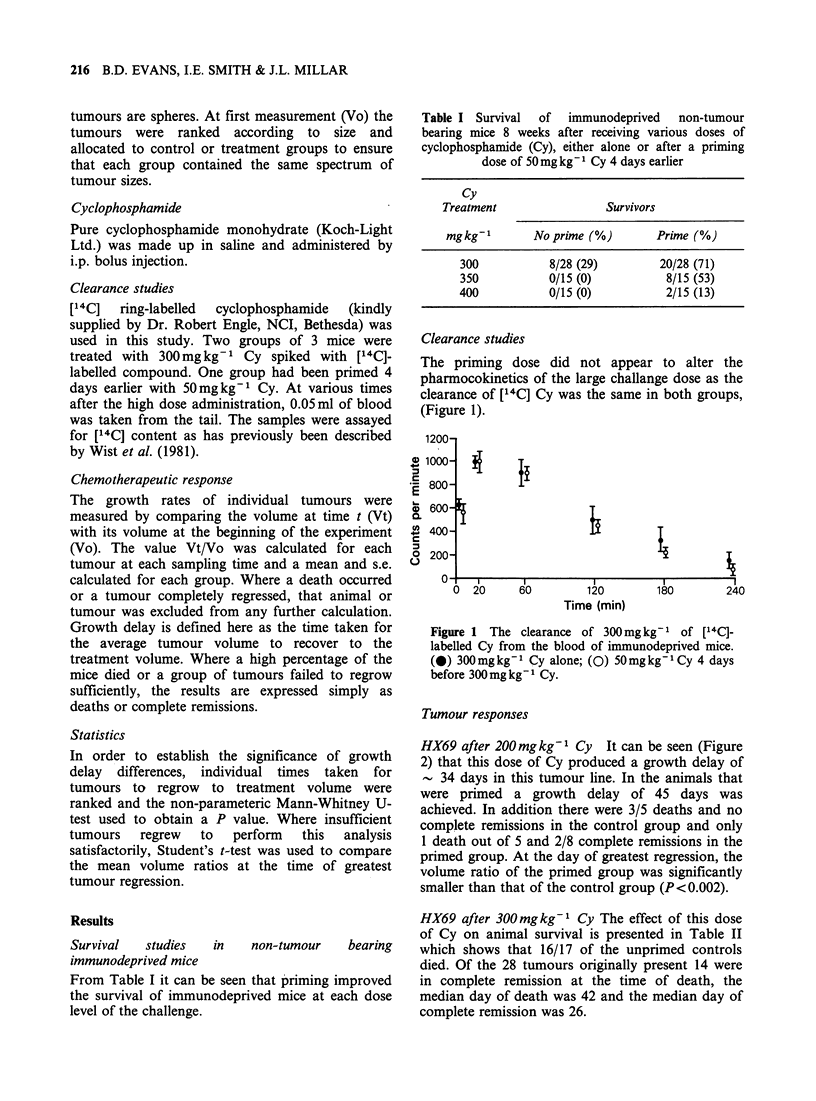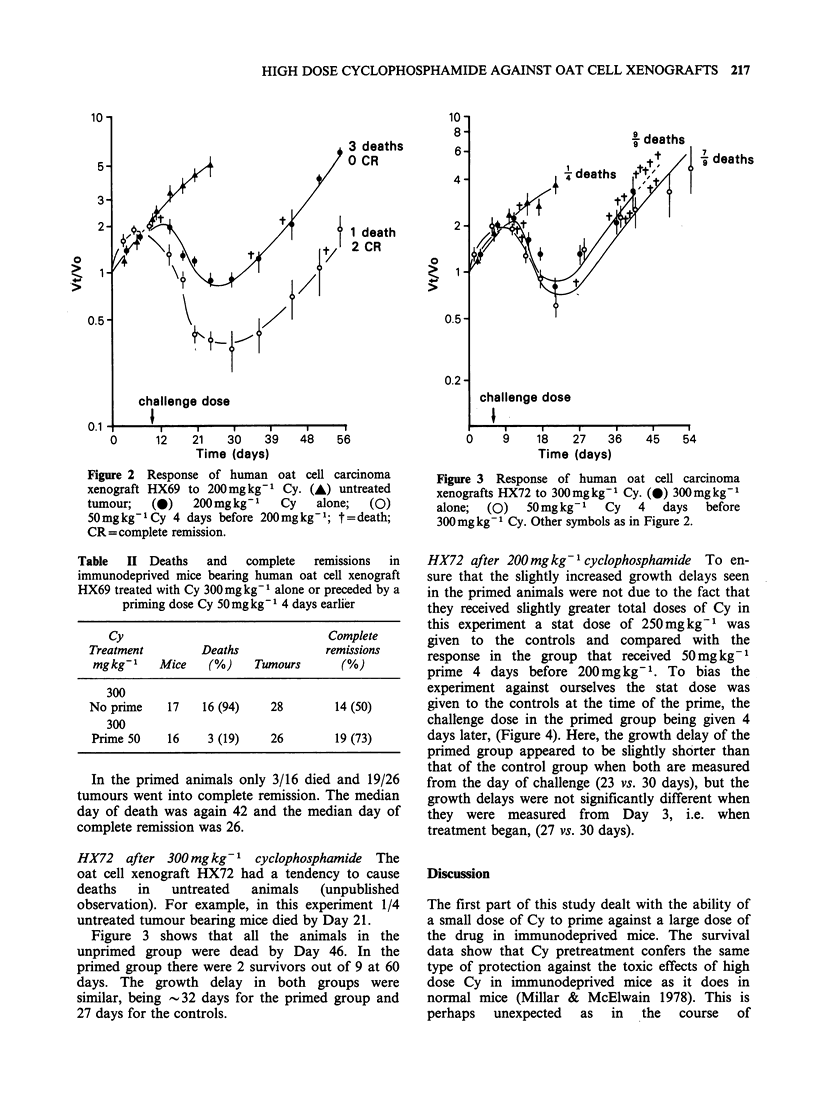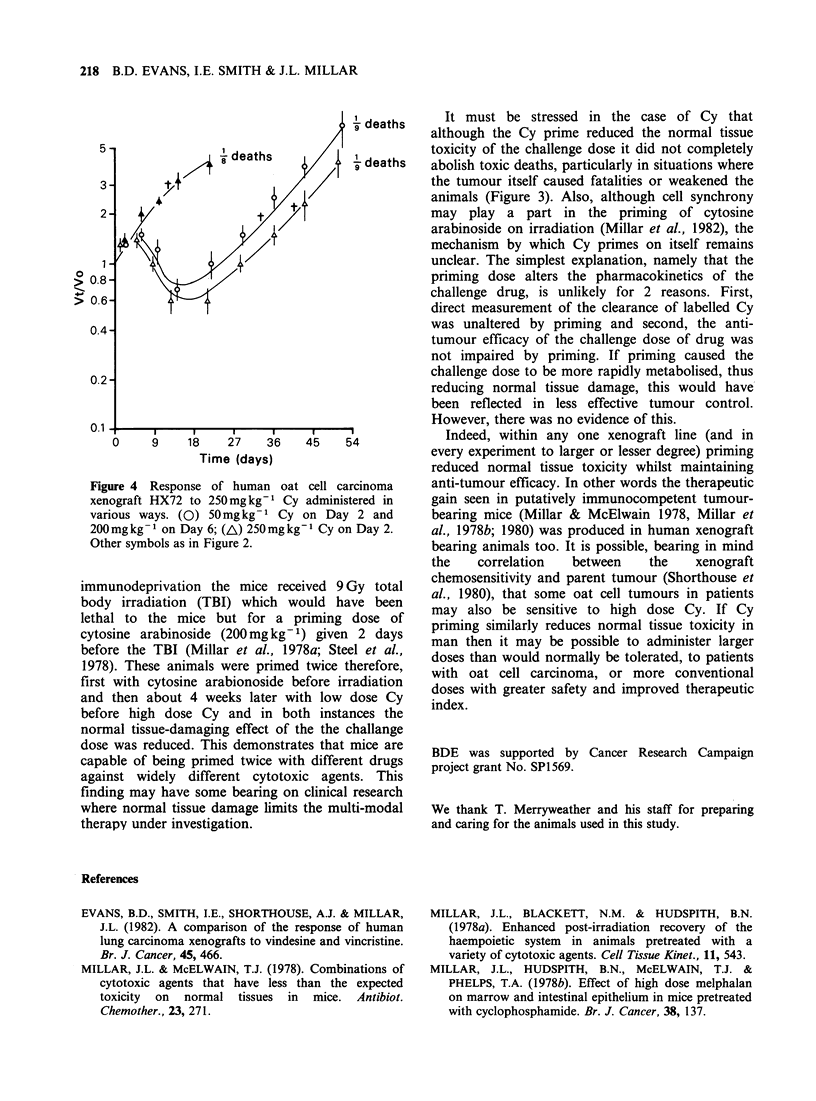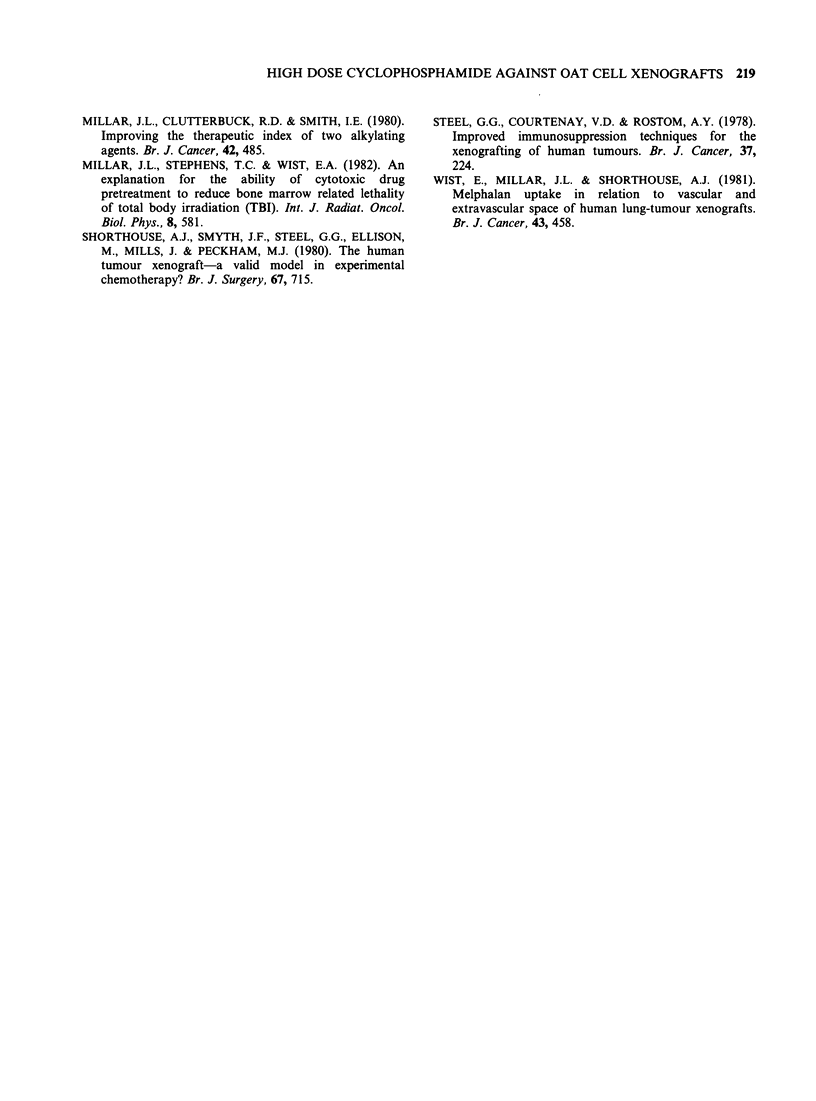# High dose cyclophosphamide treatment of human oat cell xenografts in immune deprived mice.

**DOI:** 10.1038/bjc.1983.29

**Published:** 1983-02

**Authors:** B. D. Evans, I. E. Smith, J. L. Millar

## Abstract

Immunodeprived mice survived a high, otherwise lethal dose of cyclophosphamide (Cy) provided they had been "primed" with a low dose (50 mg kg-1) of the drug 4 days earlier. These combinations were then tested on 2 oat cell xenograft lines (which are known to reproduce the chemotherapeutic responses of the parent tumours) grown in immunodeprived mice. In the treatment of the first oat cell xenograft, 200 mg kg-1 Cy produced a growth delay of 34 days in the unprimed group and 45 days in the primed group. At a dose of 300 mg kg-1 a growth delay could not be assessed in the control group as 16/17 of these unprimed mice bearing this xenograft died. However, 14/22 tumours went into complete remission in this group before death occurred. In contrast only 3/16 deaths occurred in the group of mice that were primed before receiving the same challenge dose. In these animals 19/26 tumours went into complete remission and were still completely absent when the experiment was terminated at 60 days. Using the second oat cell xenograft, 300 mg kg-1 Cy produced a growth delay of 27 days. However, at this dose level all the animals were dead by day 46. In mice which had been primed with 50 mg kg-1 Cy 4 days before the administration of 300 mg kg-1 a growth delay of 32 days was achieved and 2/9 animals were alive at day 60. This study shows that priming allows larger doses of Cy to be given to immunodeprived mice bearing human tumour xenografts than would normally be tolerated and that the priming does not alter the anti-tumour efficacy of the large challenge dose as measured by tumour growth delay or complete remission rate. As the tumours were human in origin it raises the question whether high dose cyclophosphamide therapy and priming have a role to play in the treatment of patients with oat cell carcinoma.


*Br. J. Cancer* (1983), **47**, 215–219

### High dose cyclophosphamide treatment of human oat cell xenografts in immune deprived mice

B.D. Evans, I.E. Smith & J.L. Millar*

*Royal Marsden Hospital, Fulham Road, London, SW3 6JJ & *Institute of Cancer Research, Sutton, Surrey, SM2 5PX.*

**Summary** Immunodeprived mice survived a high, otherwise lethal dose of cyclophosphamide (Cy) provided they had been "primed" with a low dose ($50\,mg\,kg^{-1}$) of the drug 4 days earlier. These combinations were then tested on 2 oat cell xenograft lines (which are known to reproduce the chemotherapeutic responses of the parent tumours) grown in immunodeprived mice. In the treatment of the first oat cell xenograft, $200\,mg\,kg^{-1}$ Cy produced a growth delay of 34 days in the unprimed group and 45 days in the primed group. At a dose of $300\,mg\,kg^{-1}$ a growth delay could not be assessed in the control group as 16/17 of these unprimed mice bearing this xenograft died. However, 14/22 tumours went into complete remission in this group before death occurred. In contrast only 3/16 deaths occurred in the group of mice that were primed before receiving the same challenge dose. In these animals 19/26 tumours went into complete remission and were still completely absent when the experiment was terminated at 60 days. Using the second oat cell xenograft, $300\,mg\,kg^{-1}$ Cy produced a growth delay of 27 days. However, at this dose level all the animals were dead by day 46. In mice which had been primed with $50\,mg\,kg^{-1}$ Cy 4 days before the administration of $300\,mg\,kg^{-1}$ a growth delay of 32 days was achieved and 2/9 animals were alive at day 60. This study shows that priming allows larger doses of Cy to be given to immunodeprived mice bearing human tumour xenografts than would normally be tolerated and that the priming does not alter the anti-tumour efficacy of the large challenge dose as measured by tumour growth delay or complete remission rate. As the tumours were human in origin it raises the question whether high dose cyclophosphamide therapy and priming have a role to play in the treatment of patients with oat cell carcinoma.

It has been known for some time that low dose cyclophosphamide (Cy) pretreatment or "priming" 4 days before high dose administration improves mouse survival in otherwise normal C57B1 mice, (Millar & McElwain, 1978; Millar *et al.*, 1980). It was also demonstrated that priming does not alter the efficacy of the large challenge dose in terms of the curability of the Lewis lung carcinoma or tumour growth delay of the FS6 fibrosarcoma both of which are syngeneic in C57B1 mice (Millar & McElwain, 1978; Millar *et al.*, 1980). The mechanism underlying these observations is not understood.

The object of this study was first to establish whether immunodeprived mice bearing human oat cell tumour xenografts would withstand high dose Cy therapy better when they had been primed 4 days earlier with low dose Cy ($50\,mg\,kg^{-1}$), and then to see if the anti-tumour efficacy of the high dose was altered by priming.

Correspondence: J.L. Miller, Institute of Cancer Research, Sutton, Surrey, SM2 5PX.

Received 23 August 1982; accepted 29 October 1982.

0007-0920/83/020215-05 $02.00

© The Macmillan Press Ltd., 1983

### Materials and methods

### *Mice*

Female CBA/lac mice were immunosuppressed by thymectomy at 4 weeks of age, followed 4 weeks later by 9 Gy whole body irradiation, (Steel *et al.*, 1978).

### *Tumours*

The 2 human oat cell xenograft lines used in these experiments, HX69 and HX72, (HX being the acronym for human xenograft), were originally established from material obtained by surgical biopsy. The tumours used in these experiments were in early passage (6–14) and had been shown by histology and chromosome analysis to have retained their human characteristics, (Shorthouse *et al.*, 1980). At the start of each experiment, tumour fragments measuring about 1–2 mm were bilaterally implanted s.c. into the flanks of these 8–10 week old mice, as described elsewhere, (Evans *et al.*, 1982). The animals were used when the tumours had reached a volume of $0.3$–$0.5\,cm^3$ calculated by the volume formula $V=\pi LD^2/6$, where V = volume, L = longest diameter and D = diameter at right angles to it. This is the formula for an oblate spheroid and compensates for the fact that not all tumours are spheres. At first measurement (Vo) the tumours were ranked according to size and allocated to control or treatment groups to ensure that each group contained the same spectrum of tumour sizes.

*Cyclophosphamide*

Pure cyclophosphamide monohydrate (Koch-Light Ltd.) was made up in saline and administered by i.p. bolus injection.

*Clearance studies*

[$^{14}$C] ring-labelled cyclophosphamide (kindly supplied by Dr. Robert Engle, NCI, Bethesda) was used in this study. Two groups of 3 mice were treated with 300 mg kg$^{-1}$ Cy spiked with [$^{14}$C]-labelled compound. One group had been primed 4 days earlier with 50 mg kg$^{-1}$ Cy. At various times after the high dose administration, 0.05 ml of blood was taken from the tail. The samples were assayed for [$^{14}$C] content as has previously been described by Wist *et al.* (1981).

*Chemotherapeutic response*

The growth rates of individual tumours were measured by comparing the volume at time *t* (Vt) with its volume at the beginning of the experiment (Vo). The value Vt/Vo was calculated for each tumour at each sampling time and a mean and s.e. calculated for each group. Where a death occurred or a tumour completely regressed, that animal or tumour was excluded from any further calculation. Growth delay is defined here as the time taken for the average tumour volume to recover to the treatment volume. Where a high percentage of the mice died or a group of tumours failed to regrow sufficiently, the results are expressed simply as deaths or complete remissions.

*Statistics*

In order to establish the significance of growth delay differences, individual times taken for tumours to regrow to treatment volume were ranked and the non-parameteric Mann-Whitney U-test used to obtain a *P* value. Where insufficient tumours regrew to perform this analysis satisfactorily, Student's *t*-test was used to compare the mean volume ratios at the time of greatest tumour regression.

### Results

*Survival studies in non-tumour bearing immunodeprived mice*

From Table I it can be seen that priming improved the survival of immunodeprived mice at each dose level of the challenge.

**Table I** Survival of immunodeprived non-tumour bearing mice 8 weeks after receiving various doses of cyclophosphamide (Cy), either alone or after a priming dose of 50 mg kg$^{-1}$ Cy 4 days earlier

| *Cy Treatment* | *Survivors* | |
|---|---|---|
| *mg kg$^{-1}$* | *No prime (%)* | *Prime (%)* |
| 300 | 8/28 (29) | 20/28 (71) |
| 350 | 0/15 (0) | 8/15 (53) |
| 400 | 0/15 (0) | 2/15 (13) |

*Clearance studies*

The priming dose did not appear to alter the pharmocokinetics of the large challange dose as the clearance of [$^{14}$C] Cy was the same in both groups, (Figure 1).

**Figure 1** The clearance of 300 mg kg$^{-1}$ of [$^{14}$C]-labelled Cy from the blood of immunodeprived mice. (●) 300 mg kg$^{-1}$ Cy alone; (○) 50 mg kg$^{-1}$ Cy 4 days before 300 mg kg$^{-1}$ Cy.

*Tumour responses*

*HX69 after 200 mg kg$^{-1}$ Cy* It can be seen (Figure 2) that this dose of Cy produced a growth delay of ~ 34 days in this tumour line. In the animals that were primed a growth delay of 45 days was achieved. In addition there were 3/5 deaths and no complete remissions in the control group and only 1 death out of 5 and 2/8 complete remissions in the primed group. At the day of greatest regression, the volume ratio of the primed group was significantly smaller than that of the control group ($P < 0.002$).

*HX69 after 300 mg kg$^{-1}$ Cy* The effect of this dose of Cy on animal survival is presented in Table II which shows that 16/17 of the unprimed controls died. Of the 28 tumours originally present 14 were in complete remission at the time of death, the median day of death was 42 and the median day of complete remission was 26.

**Figure 2** Response of human oat cell carcinoma xenograft HX69 to 200 mg kg$^{-1}$ Cy. (▲) untreated tumour; (●) 200 mg kg$^{-1}$ Cy alone; (○) 50 mg kg$^{-1}$ Cy 4 days before 200 mg kg$^{-1}$; †=death; CR=complete remission.

**Figure 3** Response of human oat cell carcinoma xenografts HX72 to 300 mg kg$^{-1}$ Cy. (●) 300 mg kg$^{-1}$ alone; (○) 50 mg kg$^{-1}$ Cy 4 days before 300 mg kg$^{-1}$ Cy. Other symbols as in Figure 2.

**Table II** Deaths and complete remissions in immunodeprived mice bearing human oat cell xenograft HX69 treated with Cy 300 mg kg$^{-1}$ alone or preceded by a priming dose Cy 50 mg kg$^{-1}$ 4 days earlier

| *Cy Treatment mg kg$^{-1}$* | *Mice* | *Deaths (%)* | *Tumours* | *Complete remissions (%)* |
|---|---|---|---|---|
| 300 No prime | 17 | 16 (94) | 28 | 14 (50) |
| 300 Prime 50 | 16 | 3 (19) | 26 | 19 (73) |

In the primed animals only 3/16 died and 19/26 tumours went into complete remission. The median day of death was again 42 and the median day of complete remission was 26.

*HX72 after 300 mg kg$^{-1}$ cyclophosphamide* The oat cell xenograft HX72 had a tendency to cause deaths in untreated animals (unpublished observation). For example, in this experiment 1/4 untreated tumour bearing mice died by Day 21.

Figure 3 shows that all the animals in the unprimed group were dead by Day 46. In the primed group there were 2 survivors out of 9 at 60 days. The growth delay in both groups were similar, being ~32 days for the primed group and 27 days for the controls.

*HX72 after 200 mg kg$^{-1}$ cyclophosphamide* To ensure that the slightly increased growth delays seen in the primed animals were not due to the fact that they received slightly greater total doses of Cy in this experiment a stat dose of 250 mg kg$^{-1}$ was given to the controls and compared with the response in the group that received 50 mg kg$^{-1}$ prime 4 days before 200 mg kg$^{-1}$. To bias the experiment against ourselves the stat dose was given to the controls at the time of the prime, the challenge dose in the primed group being given 4 days later, (Figure 4). Here, the growth delay of the primed group appeared to be slightly shorter than that of the control group when both are measured from the day of challenge (23 *vs.* 30 days), but the growth delays were not significantly different when they were measured from Day 3, i.e. when treatment began, (27 *vs.* 30 days).

### Discussion

The first part of this study dealt with the ability of a small dose of Cy to prime against a large dose of the drug in immunodeprived mice. The survival data show that Cy pretreatment confers the same type of protection against the toxic effects of high dose Cy in immunodeprived mice as it does in normal mice (Millar & McElwain 1978). This is perhaps unexpected as in the course of

**Figure 4** Response of human oat cell carcinoma xenograft HX72 to $250 mg kg^{-1}$ Cy administered in various ways. (○) $50 mg kg^{-1}$ Cy on Day 2 and $200 mg kg^{-1}$ on Day 6; (△) $250 mg kg^{-1}$ Cy on Day 2. Other symbols as in Figure 2.

immunodeprivation the mice received 9 Gy total body irradiation (TBI) which would have been lethal to the mice but for a priming dose of cytosine arabinoside ($200 mg kg^{-1}$) given 2 days before the TBI (Millar *et al.*, 1978*a*; Steel *et al.*, 1978). These animals were primed twice therefore, first with cytosine arabionoside before irradiation and then about 4 weeks later with low dose Cy before high dose Cy and in both instances the normal tissue-damaging effect of the the challange dose was reduced. This demonstrates that mice are capable of being primed twice with different drugs against widely different cytotoxic agents. This finding may have some bearing on clinical research where normal tissue damage limits the multi-modal therapy under investigation.

It must be stressed in the case of Cy that although the Cy prime reduced the normal tissue toxicity of the challenge dose it did not completely abolish toxic deaths, particularly in situations where the tumour itself caused fatalities or weakened the animals (Figure 3). Also, although cell synchrony may play a part in the priming of cytosine arabinoside on irradiation (Millar *et al.*, 1982), the mechanism by which Cy primes on itself remains unclear. The simplest explanation, namely that the priming dose alters the pharmacokinetics of the challenge drug, is unlikely for 2 reasons. First, direct measurement of the clearance of labelled Cy was unaltered by priming and second, the anti-tumour efficacy of the challenge dose of drug was not impaired by priming. If priming caused the challenge dose to be more rapidly metabolised, thus reducing normal tissue damage, this would have been reflected in less effective tumour control. However, there was no evidence of this.

Indeed, within any one xenograft line (and in every experiment to larger or lesser degree) priming reduced normal tissue toxicity whilst maintaining anti-tumour efficacy. In other words the therapeutic gain seen in putatively immunocompetent tumour-bearing mice (Millar & McElwain 1978, Millar *et al.*, 1978*b*; 1980) was produced in human xenograft bearing animals too. It is possible, bearing in mind the correlation between the xenograft chemosensitivity and parent tumour (Shorthouse *et al.*, 1980), that some oat cell tumours in patients may also be sensitive to high dose Cy. If Cy priming similarly reduces normal tissue toxicity in man then it may be possible to administer larger doses than would normally be tolerated, to patients with oat cell carcinoma, or more conventional doses with greater safety and improved therapeutic index.

BDE was supported by Cancer Research Campaign project grant No. SP1569.

We thank T. Merryweather and his staff for preparing and caring for the animals used in this study.

### References

EVANS, B.D., SMITH, I.E., SHORTHOUSE, A.J. & MILLAR, J.L. (1982). A comparison of the response of human lung carcinoma xenografts to vindesine and vincristine. *Br. J. Cancer*, **45**, 466.

MILLAR, J.L. & McELWAIN, T.J. (1978). Combinations of cytotoxic agents that have less than the expected toxicity on normal tissues in mice. *Antibiot. Chemother.*, **23**, 271.

MILLAR, J.L., BLACKETT, N.M. & HUDSPITH, B.N. (1978*a*). Enhanced post-irradiation recovery of the haempoietic system in animals pretreated with a variety of cytotoxic agents. *Cell Tissue Kinet.*, **11**, 543.

MILLAR, J.L., HUDSPITH, B.N., McELWAIN, T.J. & PHELPS, T.A. (1978*b*). Effect of high dose melphalan on marrow and intestinal epithelium in mice pretreated with cyclophosphamide. *Br. J. Cancer*, **38**, 137.

MILLAR, J.L., CLUTTERBUCK, R.D. & SMITH, I.E. (1980). Improving the therapeutic index of two alkylating agents. *Br. J. Cancer*, **42**, 485.

MILLAR, J.L., STEPHENS, T.C. & WIST, E.A. (1982). An explanation for the ability of cytotoxic drug pretreatment to reduce bone marrow related lethality of total body irradiation (TBI). *Int. J. Radiat. Oncol. Biol. Phys.*, **8**, 581.

SHORTHOUSE, A.J., SMYTH, J.F., STEEL, G.G., ELLISON, M., MILLS, J. & PECKHAM, M.J. (1980). The human tumour xenograft—a valid model in experimental chemotherapy? *Br. J. Surgery*, **67**, 715.

STEEL, G.G., COURTENAY, V.D. & ROSTOM, A.Y. (1978). Improved immunosuppression techniques for the xenografting of human tumours. *Br. J. Cancer*, **37**, 224.

WIST, E., MILLAR, J.L. & SHORTHOUSE, A.J. (1981). Melphalan uptake in relation to vascular and extravascular space of human lung-tumour xenografts. *Br. J. Cancer*, **43**, 458.